# Understanding Quality Paradigm Shifts in the Evolving Pharmaceutical Landscape: Perspectives from the USP Quality Advisory Group

**DOI:** 10.1208/s12248-021-00634-5

**Published:** 2021-10-15

**Authors:** Jane Weitzel, Horacio Pappa, Gregory M. Banik, Amy R. Barker, Elizabeth Bladen, Narendra Chirmule, Joseph DeFeo, Jennifer Devine, Steven Emrick, Taha Kass Hout, Michael S. Levy, Gugu N. Mahlangu, Barbara Rellahan, Jaap Venema, Wesley Workman

**Affiliations:** 1 USP General Chapters–Measurement and Data Quality Expert Committee, Rockville, Maryland USA; 2 United States Pharmacopeia, 12601 Twinbrook Pkwy, Rockville, Maryland 20852 USA; 3 STMatics, LLC, Philadelphia, Pennsylvania USA; 4 Eli Lilly and Company, Indianapolis, Indiana 46285 USA; 5 Symphony Tech Biologics, Bengaluru, Karnataka India; 6 Juran Inc., 160 Main St., Southington, Connecticut 06489 USA; 7 Amazon Web Services (AWS), 410 Terry Ave., North Seattle, Washington, 98109 USA; 8 Medicines Control Authority of Zimbabwe (MCAZ), Harare, Zimbabwe; 9 Amgen, 601 13th Street N.W., 12th Floor, Washington, District of Columbia 20005 USA; 10 USP General Chapters–Biological Analysis Expert Committee, Rockville, MD USA

**Keywords:** Advanced manufacturing, Analytical procedures lifecycle, Digital technologies and performance-based standards, Risk-based quality management systems, Supply chain

## Abstract

Recent changes in the pharmaceutical industry have led to significant paradigm shifts in the pharmaceutical quality environment. Globalization of the pharmaceutical industry, increasingly rapid development of novel therapies, and adoption of new manufacturing techniques have presented numerous challenges for the established regulatory framework and quality environment and are impacting the approaches utilized to ensure the quality of pharmaceutical products. Regulators, industry, and standards-setting organizations have begun to recognize the need to rely more on integrated risk-based approaches and to create more nimble and flexible standards to complement these efforts. They also increasingly have recognized that quality needs to be built into systems and processes throughout the lifecycle of the product. Moreover, the recent COVID-19 crisis has emphasized the need to adopt practices that better promote global supply chain resilience. In this paper, the USP Quality Advisory Group explores the various paradigm shifts currently impacting pharmaceutical quality and the approaches that are being taken to adapt to this new environment. Broad adoption of the Analytical Procedure Lifecycle approach, improved data management, and utilization of digital technologies are identified as potential solutions that can help meet the challenges of these quality paradigm shifts. Further discussion and collaboration among stakeholders are needed to pursue these and other solutions that can ensure a continued focus on quality while facilitating pharmaceutical innovation and development.

## INTRODUCTION

Defining expectations for medicine quality and establishing conformance to those expectations traditionally has been a joint effort involving regulators, the pharmaceutical industry, pharmacopeias, and other stakeholders. Until recently, this was a relatively straightforward exercise: quality standards were established following product approval, essentially mirroring the specifications approved by the relevant regulatory agency, and quality was determined by testing the end product. Providers and patients have had confidence in the medicines they used knowing that the final product met pharmacopeial standards and other regulatory requirements.

Over the past two decades, however, there have been dramatic and sweeping changes in pharmaceutical development and the manufacturing environment—changes which are only accelerating as we look into the future. These changes are having significant implications for how we think about pharmaceutical quality and offer both opportunities and challenges for better defining and measuring quality.

In 2019, USP formed a Quality Advisory Group to assist in exploring the changes in the pharmaceutical development and the manufacturing environment that have occurred and implications for how regulators, industry, pharmacopeias, and others collaborate to safeguard the quality of medicines. The purpose of this paper is to share the findings of the Advisory Group. More broadly, USP seeks to assist industry, regulators, and other stakeholders by creating a platform for investigating these quality paradigm shifts, and ultimately, the development and implementation of collaborative solutions.

## PHARMACEUTICAL INDUSTRY TRENDS

### Domestic to Global

In recent years, the pharmaceutical industry has become truly globalized, at all stages of the drug development process. Greater than 40% of finished drugs and 80% of active pharmaceutical ingredients sold are produced outside of the USA (1). Medicines are now developed and manufactured with a global mindset, with manufacturers focused on optimizing manufacturing across multiple production sites in countries with highly variable technical and regulatory capacities. This trend will only continue in the future, as pharmaceutical manufacturers look to a global market fueled by expansion in “pharmerging” countries (e.g., China, Brazil, Russia, India, and South Korea) (2) as well as aging demographic trends in developed countries (3).

Recently, the COVID-19 pandemic has exposed numerous vulnerabilities and challenges created by the globalization of the pharmaceutical industry. While concern over the lack of security and robustness of the supply chain has been growing over the past two decades, the impact of the COVID-19 pandemic has made it starkly apparent that this is a critical issue that needs to be promptly addressed. It is now recognized more than ever the need to establish and evolve quality standards to reflect the global nature of the pharmaceutical supply chain to help ensure quality medicines and avoid potentially harmful consequences. As a result of this globalization:

- There is a need for greater harmonization of quality standards and requirements, so that manufacturers are not faced with conflicting and/or duplicative requirements across countries that add cost without providing value.
- The pharmaceutical supply chain has become increasingly complex due to operations globalization, local sourcing, customer accommodation (i.e., variety and customization of products), and managing omnichannel supply chains amid technology turnover and company mergers (4). Globalization specifically has introduced additional points of vulnerability in the supply chain and stakeholders are exploring a number of track and trace (TnT) type of solutions to help (e.g., blockchain) (5).
- A key challenge in a globalized market is how to safeguard the quality and consistency of medicines without increasing the barriers to access in less developed countries which may lack the resources or technology to meet or enforce stringent standards.

Quality expectations need to be managed in a way that facilitates efficient drug development, manufacturing, and distribution on a global scale, while ensuring that providers and patients in all parts of the world can obtain quality medicines.

### New Therapies and Modalities

A second major pharmaceutical industry trend has been the introduction of increasingly complex and sophisticated therapies and treatment modalities.

- In a review article examining the drug development pipeline in 2017, approximately three-quarters (74%) of clinical-phase projects were potentially first-in-class (6).
- Combination products are becoming increasingly common, including drug-device and diagnostic-drug products that allow medicines to be better targeted and delivered.
- Digital therapeutics, which use technology to help manage, monitor, and prevent illness, are rapidly being introduced and implemented both as an alternative and supplement to conventional medicines.
- Personalized or precision medicine, which can tailor products to individual patients based on genetic characteristics and predicted outcomes, is a promising and quickly growing area of cell and gene therapies.
- Advances in engineering and software technologies have transformed the drug development process, from gene sequencing, process engineering, and analytical technologies to data modeling and analytics. Ensuring quality standards in this fast-paced environment with rapid technological advances will require close alignment with industry, regulators, and other stakeholders.

These new advanced therapies and modalities require new thinking about how quality can be evaluated and determined. Traditional quality expectations—identity, strength, quality, and purity—may need to be re-defined for new modalities to establish consistent and appropriate expectations for these products to help ensure their quality.

### New Manufacturing Approaches

Driven by goals such as increased efficiency, a desire for a smaller environmental impact, reduced risk of drug product quality issues, and shorter time to production, the pharmaceutical industry is starting to move to new and alternative technological and manufacturing approaches.

- Pharmaceutical manufacturing is transitioning from the traditional “batch” process—in which finished products are made in an intermittent series of steps—to more efficient approaches and control strategies for manufacturing drug substances and drug products (e.g., continuous manufacturing, “on-demand” manufacturing, role of automation) (7).
- Improved data and analytical technologies are available to measure more components and intermediates; each with increasingly more accuracy.
- *In silico* modeling approaches, such as digital twins, use of electronic-data capture, analysis, and monitoring systems are being used.
- New manufacturing trends continue to emerge such as 3D printing and single-use/disposable equipment that allow modularized manufacturing to scale up capacity with potentially no changes to manufacturing facilities or equipment (8).

As technology continues to advance, these novel approaches to manufacturing will continue to gain traction in the industry. It will be critical to have regulatory pathways that provide clear expectations for how to introduce and support new technologies to facilitate timely implementation. Establishment of guidance and best practices for adoption of these technologies will also be necessary to facilitate their efficient and economical adoption, which in turn will support and encourage broader adoption and improve quality.

## QUALITY PARADIGM SHIFTS

Associated with these major industry trends, and in many cases resulting from them, are fundamental and interrelated shifts in how industry, regulators, and standards-setting bodies are approaching and assessing quality.

### Paradigm Shift #1: from Compliance-Driven to Integrated Risk-Based Approaches

In the past, the need to show regulatory compliance to current good manufacturing practices (CGMP) (9, 10), including federal regulations regarding testing and release for distribution (11), was a key driver of product decisions and created a focus on end-product testing, with a binary pass/fail result (12). There is a recognition that the goal is not compliance per se*,* but rather the ability to ensure quality as products move through development and manufacturing through the use of mature quality systems and metrics. The binary pass/fail result is enhanced and supported with increased product knowledge and understanding. As a result:

- The pharmaceutical industry and regulators are exploring pathways toward identifying and mitigating risks throughout the lifecycle of medicines (13).
- Industry increasingly is employing statistical and scientific techniques to analyze and address risk, to help prevent failures instead of detecting them after the fact.
- Predictive analytics are being used to better understand and anticipate areas of risk and vulnerability.
- The pharmaceutical industry is using science and risk-based approaches to establish patient-centric control strategies.

As data aggregation and analytical capabilities continue to expand, the ability to better identify risk and target opportunities for quality improvement will also continue to grow and should be leveraged. For adoption of risk-based control strategies to be successful, industry needs to demonstrate the rigor and applicability of these strategies, while regulatory pathways and guidelines need to provide flexibility and clear expectations for implementation of such approaches.

### Paradigm Shift #2: from Prescriptive Approaches to Flexible Performance and Outcome-Based Standards

Given the increasing rate of innovation in pharmaceutical therapies and manufacturing methods, as well as the need to accommodate a diverse global marketplace, overly prescriptive approaches to determining product quality may impede innovation and slow patient access to needed new therapies.

- Standards should accommodate and facilitate development of new products and therapies, and lower barriers to innovation and access.
- Standards need to account for wide variations in technological capabilities and resources of industry and regulators globally.
- Standards should be designed to offer stakeholders options to achieve the same or better-quality products by focusing on performance and outcome-based standards.

The challenge is to ensure that even as standards become more flexible, they will continue to provide the same assurance of medicine quality and consistency for providers and patients in all parts of the world. Moreover, to be effective, these approaches should be implemented as a coordinated effort between regulators around the globe via harmonization and alignment of requirements and standards.

### Paradigm Shift #3: from Testing Products to Building Product Quality

There is an increasingly prevalent view that quality needs to be built into systems and processes instead of “tested in” to products at the end (14).

- The pharmaceutical industry and regulators are increasingly embracing Quality by Design (QbD) approaches, which comprise a system of knowledge that enables pre-defined attributes for process control and product quality as outlined in ICH guidelines Q8–11. Integrating QbD into the development and manufacturing process helps ensure consistent product quality by understanding and controlling formulation and manufacturing variables and critical quality attributes (15).
- QbD and process analytical technology (PAT) can enable real-time process adjustments within predefined ranges of quality and in a manner that can be applied across products and facilities. These in-process control decisions can provide an increased level of quality assurance compared to traditional end-product testing and allow for real-time release testing of drug products (16).
- There is a recognition that quality needs to extend beyond systems and processes to the organization itself. Increasingly, pharmaceutical manufacturers are seeking to establish a “culture of quality” in which all employees feel responsible for quality (17, 18).

The shift toward QbD and an organization-wide commitment to quality is already underway, and compendial and regulatory approaches must continue evolving to support and help advance this transformation.

### Paradigm Shift #4: Need to Ensure Supply Chain Resilience in a Quality Environment

The globalization of the pharmaceutical industry and supply chain has benefited patients by providing increased access to medicines—particularly low-cost generics—both in the USA and around the world. Unfortunately, this shift leads to a global supply chain that has numerous vulnerabilities and increased risks to the quality of drugs.

Specifically, one of the main risks created by the global supply chain is an increase in the potential for drug shortages of many critical medicines. The complexity of the global supply chain can create logistical and regulatory challenges which make it difficult for drug manufacturers to quickly ramp up production in response to increases in demand (19). Additional vulnerabilities are driven by greater complexity of the upstream supply chain and include reliance on “just-in-time” manufacturing, lack of redundancy in suppliers, increased outsourcing of ingredients, and even final product manufacturing. In addition, there is a lack of transparency of the supply chain, which can limit the ability of stakeholders to take mitigative action before it is too late. All of these risks can contribute to a supply chain that lacks resilience and the ability scale-up to meet demand or absorb supply shocks. In addition to increased risk of drug shortage, the global nature of the market has also increased the risk of adulterated raw materials and finished products entering the market and has provided opportunities for data fraud (20).

Recently, the COVID-19 pandemic brought these vulnerabilities into sharp focus, leading to calls to bring back domestic manufacturing of products and increase the diversity and redundancy of the supply chain (21). These solutions are likely to be politically popular, but ultimately, market pressure will continue to encourage overseas production of pharmaceuticals and their components to areas with the lowest manufacturing costs.

More fundamental solutions are needed to ensure a resilient supply chain that has the capacity to absorb the shocks of acute disruptions and ensure the quality of raw materials, drug components, and finished drug products. Creation of a framework that incentivizes quality, supply chain robustness, and to sufficiently address the challenges of the new global supply chain could be achieved by:

- Development of standards to help ensure data integrity and provide the foundation needed to facilitate effective remote auditing and inspections.
- Development of standards and best practices that support supplier qualification and improved evaluation of raw materials.
- Adoption of guidelines and best practices that encourage manufacturers to conduct risk assessments to determine potential weaknesses in their supply chain, such as relying on a single source for raw materials and intermediates or utilization of “just-in-time” manufacturing, and development of corresponding solutions to mitigate these risks (22).
- Increased harmonization and alignment of standards between regulatory agencies and pharmacopeias around the globe.
- Adoption of methods and standards for tracking of drug components throughout the drug development process from raw materials to delivery of finished drug products.

## EFFORTS TO ADDRESS PARADIGM SHIFTS

While it is useful to understand and recognize the quality paradigm shifts in the evolving pharmaceutical landscape, the question remains as to how these changes should be addressed. Beyond just recognizing these issues, it is much more challenging to determine the consequent transformations that need to occur to address these rapid changes and meet the diverse needs of patients and industry. While industry and science evolve organically, subsequent changes to guidance, standards, and regulations require purposeful action and periodic evaluation to ensure they are aligned with contemporary and future needs. If standards and regulations fail to evolve to meet these needs, they may become increasing detrimental and hinder rather than support improvements needed to ensure quality.

Regulators, industry, academia, pharmacopeias, and other stakeholders have recognized the need for this evolution and are pursuing new regulatory and standards approaches that will help ensure quality while facilitating access to affordable as well as novel therapies. Organizations such as the National Institute for Pharmaceutical Technology and Education (NIPTE), the American Association of Pharmaceutical Scientists (AAPS), and the Product Quality Research Institute (PQRI) reflect broad collaborative efforts among these stakeholders to explore and advance emerging regulatory science topics relating to the quality and efficiency of pharmaceutical research, development, and manufacturing. The US Food and Drug Administration (FDA) has been working to find ways to accelerate and support industry adoption of advanced manufacturing technologies, and recently entered a memorandum of understanding (MOU) with the National Institute of Standards and Technology (NIST) to further this work (23).

As an outcome of its Pharmaceutical Quality for the twenty-first Century Initiative, the FDA recently introduced a new Quality Maturity Model (QMM) (24). The FDA’s interest QMM builds on ICH Guidance Q10: *Pharmaceutical Quality System*, which moves beyond CGMP and traditional quality management systems to the notion of an effective pharmaceutical quality system that facilitates innovation and continuous improvement throughout the entire product lifecycle. Like Q10, QMM emphasizes management’s essential responsibility for establishing a company-wide commitment to quality and investing in people and resources. QMM recognizes that today’s complex and dynamic pharmaceutical environment requires a multi-faceted and proactive approach to quality that includes risk management, predictive analytics, robust quality metrics, and a focus on continually improving performance and outcomes.

USP’s Quality Advisory Group has identified two fundamental solutions that can complement these efforts and help advance the transformation of quality frameworks. These are the broad adoption of the Analytical Procedures Lifecycle (APL) approach and increased utilization of digital technologies. The APL approach, which includes Analytical Quality by Design (aQbD), is being developed by organizations such as the *United States Pharmacopeia* (USP) and *British Pharmacopoeia*. In several *Stimuli Articles*, USP has presented examples of how QbD approaches could be used to support flexible yet robust strategies for analytical procedure lifecycle development. (25–33). Recently, in September 2020, USP also proposed a new general chapter 〈1220〉 Analytical Procedure Life Cycle in the *Pharmacopeial Forum* (33). The APL approach is based on and focuses on the reality that an analytical procedure must be demonstrated to be fit for its intended purpose and provides mechanisms to move from prescriptive to more flexible approaches. The purpose of applying lifecycle principles to analytical procedures is to holistically align analytical procedure variability with the requirements of the product to be tested and to improve the reliability of the procedure by understanding, reducing, and controlling sources of variability. Enhanced understanding of variables that affect the performance of an analytical procedure provides greater assurance that the quality attributes of the tested product can be reliably assessed. The APL approach provides a framework for defining the criteria for and development of an analytical procedure that meets the acceptance criteria. The procedure then becomes part of a continuous verification cycle to demonstrate that it meets the predefined criteria over the life of the procedure.

In addition to the APL approach, improved data management and increased use of digital technologies could also provide many potential solutions which could allow industry and regulators to adapt to the current paradigm shifts. Some of these advances in the digital space, such as assisted intelligence and machine learning, are driving paradigm shifts on their own. Currently, only the surface has been scratched of the potential benefits that digital technologies could provide if fully utilized and integrated throughout the pharmaceutical manufacturing process.

Applications of improved data use could include embedding of standards content software that defines methods and interfaces with lab instrumentation. Likewise, establishment of systems to ensure data integrity and automatic transfer to regulatory agencies could allow for added trust between industry and regulators. Adoption of data integrity and data sharing along with other quality processes could be incentivized by allowing for reduction in inspections and providing for streamlined approval of products. This could benefit manufacturers who establish mature quality management capabilities and allow regulatory agencies to focus their efforts on facilities at greater risk for quality issues. A step in this direction is FDA’s Knowledge-aided Assessment and Structured Application system (KASA), which uses structured data and information capture, predefined rules, and algorithms, and computer-aided analyses improve the quality, efficiency, consistency, and objectivity of FDA’s regulatory actions by evaluating risks in a more systematic fashion. The FDA’s commitment to KASA represents a significant regulatory paradigm shift, leveraging digitalization and structured data for improved access to historical data (e.g., drug flings) related to similar products already approved on the market, more objective risk assessment, and greater consistency in decision-making by regulators who are reviewing an ever-increasing number of filings (34).

Moreover, increased use of applications such as artificial intelligence and predictive analytics can provide tools that could allow for identification of potential quality risks before they even occur. For example, predictive analytics could alert a formulator to the potential presence of a previously unexpected impurity, preventing potential product recalls that could have a negative impact on the global drug supply and public health.

To realize the full potential of digital solutions, standards will be needed to establish parameters that would allow for data sharing and interoperability among various digital platforms, including analytical equipment, software platforms that analyze process/instrumentation data, and risk management platforms used by regulators that would rely on these data. Given the global nature of the pharmaceutical market, even greater benefits could be gained if this interoperability can be facilitated among regulatory agencies around the globe.

## CONCLUSION

Changes in the pharmaceutical industry are causing fundamental shifts in traditional quality paradigms. Successfully navigating through these shifts will require new and innovative thinking, as well as greater collaboration across an increasingly global and diverse set of stakeholders. This includes not just regulators, industry, and pharmacopeias, but also new stakeholders such as data technology companies, who can help harness data and analytics to better understand and drive quality.

The adoption of more flexible, agile, and iterative approaches to public standard development and delivery will also be required. Historically, standards-setting has been a static and reactive process. Given the rapid rate of change in products and technologies, standards-setting bodies and regulators will need to employ more proactive and nimble ways of introducing, evolving, and adopting standards.

Working together, with a shared recognition of both the opportunities and challenges created by this rapidly evolving environment, we can develop approaches that help ensure a continued focus on quality and continuous improvement while supporting and facilitating important advances in therapies and technologies. Increased adoption of an Analytical Procedures Lifecycle Approach and an increased use of digital technologies are just two examples of potential broad solutions that could help achieve these goals.

Further dialog and engagement with industry, regulators, and other stakeholders are needed to explore these avenues and identify additional approaches that could be utilized to meet the challenges of the current paradigm shifts. USP and its Quality Advisory Group are committed to collaborating with stakeholders to develop the best solutions to these pressing issues.^1^

## Footnotes

1 Please submit any input on the perspectives presented in this paper to Horacio Pappa, Director, General Chapters, United States Pharmacopeia, email hp@usp.org.
